# Comparative Evaluation of the Effectiveness of Using Quinoa Grain (*Chenopodium quinoa* Willd.) with High and Low Saponin Content in Broiler Chicken Feeding

**DOI:** 10.3390/ani15172574

**Published:** 2025-09-02

**Authors:** Artem Yu. Zagarin, Aleksandra V. Shitikova, Marina I. Selionova, Sergey V. Akchurin, Marianna Yu. Gladkikh

**Affiliations:** 1 Department of Animal Breeding, Genetics and Biotechnology, Institute of Animal Science and Biology, Russian State Agrarian University—Moscow Timiryazev Agricultural Academy, 49 Timiryazevskaya Str., Moscow 127434, Russia; selionova@rgau-msha.ru (M.I.S.); marianna.gladkikh@rgau-msha.ru (M.Y.G.); 2 Department of Plant Production and Meadow Ecosystems, Institute of Agrobiotechnology, Russian State Agrarian University—Moscow Timiryazev Agricultural Academy, 49 Timiryazevskaya Str., Moscow 127434, Russia; plant@rgau-msha.ru; 3 Department of Veterinary Medicine, Institute of Animal Science and Biology, Russian State Agrarian University—Moscow Timiryazev Agricultural Academy, 49 Timiryazevskaya Str., Moscow 127434, Russia; sakchurin@rgau-msha.ru

**Keywords:** non-traditional feeds, poultry feeding, animal nutrigenomics, gut health

## Abstract

The aim of this study was to conduct a comparative analysis of the effects of native quinoa grain with a high saponin content and quinoa grain subjected to preliminary saponin removal with low saponin content on growth, meat quality, biochemical blood composition, and the expression of genes related to muscle growth, gut health, and nutrient transport in broiler chickens. The control group of chickens received a standard diet. The SAP group feed contained quinoa grain without saponin removal (saponin level—5.20%) at 3% of the “Starter” feed mass and 5% of the “Grower” and “Finisher” feeds, maintaining the same nutritional values as the control group. The SAP-FREE group feed contained quinoa grain that was pre-treated to remove saponins by washing with water for 60 min at a temperature of 50 °C (saponin level—0.24%) in the same amount as the SAP group. The research results indicated certain advantages of unprocessed quinoa grain in relation to saponin content. Specifically, in the SAP group, the broiler performance index was at the same level as the control, while the SAP-FREE group had a high mortality rate (10%), resulting in a performance index that was 23.82 units lower than the control. The use of quinoa grain with high saponin content promoted better development of thigh muscles by 9.6% compared to the control (*p* = 0.008) and increased yields of wing, neck, and back muscles by 2.9 abs.% (*p* = 0.007) compared to the use of purified quinoa grain. The fat yield decreased by 1.7 abs.% (*p* = 0.015) with saponin-free quinoa compared to the control and by 2% (*p* = 0.008) compared to the high saponin group, making this feeding system viable for producing dietary meat. Upon stopping the feeding of purified quinoa, chickens showed a 34.0% increase in AST activity (*p* = 0.019) and a 15.7% increase in creatinine levels (*p* = 0.008), likely indicating intensified protein metabolism upon cessation of the inhibiting factor of purified quinoa. Molecular genetic studies revealed a 1.6-fold increase in IGF1 gene expression (*p* = 0.014) in breast muscle and a 69.12-fold increase (*p* = 0.010) in AvBD9 in the cecum due to high-saponin quinoa grain, while purified quinoa increased GHR gene expression by 3.29 times (*p* = 0.039) in breast muscle and decreased IRF7 activity to 2^−ΔΔCT^ = 0.54 (*p* = 0.017). The expression of transporter protein genes decreased to low or undetectable levels, indicating the presence of anti-nutritional factors and the need for further research on feeding quinoa with the addition of proteases. Thus, high-saponin quinoa grain, unlike purified quinoa, positively influences gut health and bird survival, maintaining performance levels similar to the control, suggesting the feasibility of using unprocessed quinoa in poultry nutrition, thus avoiding additional costs in feed preparation.

## Simple Summary

Increasing production volumes in modern poultry farming demand the search for new unconventional sources of nutrients that serve as alternatives to the most commonly used feed components. Quinoa grain is of considerable interest, as this crop is undemanding and has a relatively high yield and nutritional value. However, quinoa contains saponins, which are characterized as anti-nutritional factors but also possess a number of biologically active beneficial properties. Removing saponins requires additional resources and costs, which is why this study was conducted. It focuses on a comparative analysis of feeding broiler chickens with quinoa grain that has been pre-treated to remove saponins versus untreated quinoa grain with a high (native) level of saponins. The results indicated some advantages for the second option. Therefore, it is advisable to use quinoa grain in poultry nutrition without prior removal of saponins, allowing for cost savings in feed preparation.

## 1. Introduction

It is known that a key source of exchange energy in the diet of poultry primarily comes from corn, while protein is sourced from soybean meal. However, the dynamic development of the poultry industry, driven by global population growth, urbanization, and rising incomes in developing countries, leading to increased demand for affordable sources of animal protein, energy, vitamins, and minerals, has heightened the need for feed raw materials and may lead to a shortage of traditional feed resources [1,2]. Therefore, the search for unconventional and less expensive nutrient sources and research into their use in poultry feeding is crucial. The search for new components in poultry feed not only addresses the need for alternatives to traditional raw materials but also enhances productivity, health, and functional feeding of poultry [3,4,5]. At the same time, the key factors limiting the use of non-traditional, less expensive sources of energy and protein in poultry nutrition are high fiber content; lower bioavailability of proteins, vitamins, and minerals; less effective digestion and absorption of nutrients; and the presence of antinutritional factors. These issues can negatively impact the productivity level and health status of the poultry [6,7].

As an example of a non-traditional crop for the fodder industry, quinoa (*Chenopodium quinoa* Willd.) can be considered. Quinoa is a plant originating from South America, characterized by several advantages, including high adaptability to various environmental conditions, such as low temperatures, mountainous terrain, drought, and a wide range of soil pH [8]. Previous studies have demonstrated the possibility of cultivating this crop in the European part of Russia, achieving a yield level of 1.65–4.23 t/ha depending on the variety, with a high protein content in grain ranging from 12.5% to 14.0% [9].

Thus, quinoa has high potential not only as a food product for people [8] but also as a feed for farm animals, and this is of particular importance for regions with less favorable conditions for growing other grains [10]. However, quinoa grain contains saponins, the primary secondary metabolites present in quinoa, mainly located in the outer layer of the seeds. As mentioned earlier, saponins are considered anti-nutritional factors in cereals. Therefore, methods have been developed to reduce saponin concentrations in quinoa grain [11]. In this case, the question arises of the feasibility of using different variants of this crop in poultry nutrition to find the optimal one. Firstly, any mechanical methods of processing quinoa grain and additional selection activities to remove saponins entail the need for additional infrastructure and financial resources [10]. Secondly, despite the negative qualities of saponins, they also exhibit beneficial biologically active properties, such as antioxidant, analgesic, immunostimulating, antimicrobial, antiviral, and cytotoxic activity; anti-inflammatory and hemolytic action; improved absorption of certain minerals and vitamins; and stimulation of growth [8,11], which can have a positive effect on poultry productivity levels.

The literature provides information on the use of quinoa grain in the diet of broiler chickens [12,13,14]. However, there is a lack of studies comparing the effectiveness of feeding different variants of quinoa, either containing or free from saponins, to poultry, which highlights the novelty of this research. Our hypothesis is that it is advisable to use quinoa grain unprocessed for saponins in the diet of farm poultry, particularly broiler chickens, in order to avoid additional economic costs associated with washing quinoa or other cleaning methods. Supporting this hypothesis is the fact that birds have significantly fewer bitter taste receptors compared to mammals [15], making them less sensitive to the presence of saponins in quinoa.

Thus, the goal of this study was to conduct a comparative analysis of the effects of two quinoa grain variants. One contained saponins in their native amounts, while the other was treated to remove saponins and had a low level of these substances. The analysis focused on growth, meat productivity, biochemical blood composition, and the expression of genes related to muscle growth, gut health, and nutrient transport in broiler chickens.

## 2. Materials and Methods

The study was reviewed and approved by the Bioethics Committee of the Russian State Agrarian University—Moscow Timiryazev Agricultural Academy (protocol No. 25 dated 28 September 2024).

### 2.1. Birds and Experimental Design

The scientific experiment on poultry was conducted from 30 September to 5 November 2024, in the vivarium of the Russian State Agrarian University—Moscow Timiryazev Agricultural Academy (Moscow, Russia) in accordance with the Recommendation of the Eurasian Economic Commission Board dated 14 November 2023, No. 33 “On Guidelines for Working with Laboratory (Experimental) Animals in Preclinical (Non-Clinical) Research”. The object of the study was broiler chickens of the Smena 9 cross (the cross was created based on lines of Cornish and Plymouth Rock breeds). The chickens were purchased from the breeding and genetic center “Zagorsk Experimental Breeding Farm” (Sergiev Posad, Russia) on the hatching day. The average live weight of the chick batch was 45.6 ± 0.28 g. Before the start of the research, all the poultry were vaccinated against Gumboro and Newcastle diseases at the breeding and genetic center. All day-old chickens were examined by a full-time veterinarian from the breeding and genetic center before the start of the experiment, and no diseases or sharp deviations from the normal clinical state of health were noted. A total of 150 chickens were divided into 3 groups of 50 chickens each using a balanced group analogy method considering live weight and overall development. The ratio of cockerels to hens in the groups was randomized, as most poultry farms raise broiler chickens without sex separation. Both males and females were involved in weighing and live weight analysis; however, at the end of the rearing period, live weight and carcass quality were assessed taking sex into account, while biological studies (blood biochemical composition and gene expression) were conducted using only males. Thus, on the final day of rearing broiler chickens, a fasting period lasting 10 h was conducted to empty the chicken’s gastrointestinal tract. From each group, 3 males and 3 females with an average live weight established in the group based on weighing results were selected (a total of 18 chicks (9 cockerels and 9 hens) were selected from three groups). The broilers were slaughtered and anatomically dissected. The method of electrostunning was used. The conditions for euthanizing the animals complied with the Recommendations of the Eurasian Economic Commission Collegium from 14 November 2023, No. 33 “On the Guidelines for Working with Laboratory (Experimental) Animals in Preclinical (Non-Clinical) Research”. The sample size was selected in accordance with the Methodology for Conducting Scientific and Production Research on Feeding Agricultural Poultry, developed by the All-Russian Poultry Research Institute. The study was preliminary in nature, which limited the number of animals involved.

The first group of broiler chickens, which served as a control (CON), was fed the main diet throughout the entire rearing period, which consisted of three-phase complete feeds (“Starter” (0–10 days), “Grower” (11–22 days), and “Finisher” (23–36 days)). Native (uncrushed) quinoa grain was added to the feed of the chickens in the experimental groups (*Chenopodium quinoa* Willd.). In the second group, which was an experimental SAP, quinoa grain of the Cherry Vanilla variety (USA selection) was added to the poultry feed in the amount of 3% of the “Starter” feed mass and 5% of the “Grower” and “Finisher” feed mass without preliminary grain treatment from saponins. In the third SAP-FREE group, native quinoa grain was added to the feed in a similar manner, from which saponins were preliminarily removed by washing.

The washing time was 60 min at a water temperature of 50 °C [16]. The saponin content of quinoa grain before and after processing was determined by high-performance liquid chromatography–mass spectrometry using an Ultimate 3000 liquid chromatography system (Thermo Fisher Scientific, Waltham, MA, USA) with preliminary grinding of the grain and extraction of saponins using methanol. The initial saponin content of quinoa grain was 5.20%. After washing treatment, the saponin content of peeled seeds was 0.24%.

This study is a preliminary one; therefore, the level of grain input into the compound feed was selected for the first time. However, when preparing the study, we were guided by the results of a previously published study [14]. For the same reason, we decided to use uncrushed quinoa grain to study the effect of poultry consumption of the native form of a non-traditional feed product on biological indicators and productivity of broiler chickens, since saponins are mainly contained in the outer layer of the seeds of this plant [11]. Five days before slaughtering broilers, quinoa was excluded from the diet to avoid the negative impact of saponins on the taste of poultry meat.

Broiler chickens were kept in a separate box of the vivarium in three-tiered cage installations. When growing the chickens, the key zoo-hygienic parameters recommended by the creators of the Smena 9 cross were implemented [17]. The following microclimate conditions were provided in the premise: ambient temperature of 31–33 °C during the first 7 days of rearing, 24–30 °C from 8 to 21 days, and 18–23 °C from 22 to 36 days and relative air humidity of 40–60% during the first 7 days of the experiment and 60–70% from 8 to 36 days. Based on the daily monitoring of thermometer and hygrometer readings, the THI (temperature–humidity index) value was calculated using the formula proposed by Tom [18] and refined by Mader et al. [19]:

THI = 0.8 × Td + (RH/100) × (Td − 14.4) + 46.4(1)

where Td represents the dry bulb temperature (°C), and RH represents the relative humidity of the air (%).

**Figure 1:**
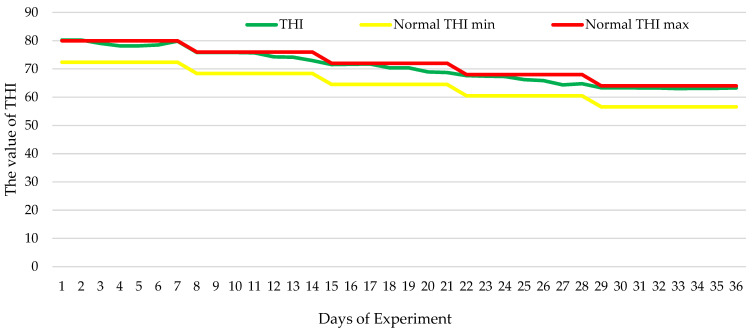
Dynamics of the temperature–humidity index during the experiment (normal THI min—minimum allowable THI values ensuring thermal comfort for broilers; normal THI max—maximum allowable THI values ensuring thermal comfort for broilers [20]).

The THI values were within the thermoneutral zone, occasionally approaching the upper limit [20] (Figure 1). Thus, no heat stress occurred under the experimental conditions.

The power mode during the experiment was constant light on the first day of rearing, 23 h of light and 1 h of darkness at the age of 1–7 days, 4 cycles of 5 h light and 1 h of darkness at the age of 8–34 days, and 23 h of light and 1 h of darkness at the age of 35–36 days. Fluorescent lamps with white lighting were used at intensities of 25 lux (0 days), 20 lux (1–7 days), and 10 lux (8–36 days). The floor area in each individual section of the cell was 65 cm × 70 cm. The young poultry were housed with a final density of rearing of 21 heads per 1 m^2^ (476 cm^2^ per 1 individual). The chickens were placed evenly. The experiment was conducted using an ad libitum feeding and watering regime. The chickens were fed manually in the morning using bunker and trough feeders depending on the age of the poultry. The chickens were watered using vacuum and nipple drinkers depending on the age of the poultry. Paper was used as bedding during the early growth period. The duration of the scientific experiment on poultry was 36 days. The use of veterinary medicines was not applied during the experiment.

### 2.2. Broiler Chicken Nutrition

During the experimental period, complete-phase compound feeds in the form of granules were used: up to 10 days of age—“Starter”, from 11 to 22 days—compound feed “Grower”, from 23 to 35 days—compound feed “Finisher”. With the additional introduction of quinoa grain into the compound feed, the nutritional value and the main indicators of the chemical composition of the feed (crude protein, crude fiber, calcium, phosphorus, lysine, and methionine + cystine) were balanced by changing the ratio of the components of the compound feed to maintain the control and experimental groups, primarily by reducing the proportion of corn and soybean meal. The chemical composition and the level of metabolic energy of the compound feed corresponded to the recommended feeding indicators for broiler chickens of the Smena 9 cross [17] (Table 1). Quinoa in its native form was introduced into the composition of ready-made granulated compound feed by gradually diluting and mixing it.

Table 2 shows the amino acid composition of the quinoa grain used.

### 2.3. Growth and Meat Productivity of Broiler Chickens

During the experiment, individual weightings of all experimental broiler chickens were carried out at the ages of 0, 7, 14, 21, 28, and 36 days. All chicks in each group were weighed. Individual live weight data of the chicks were included in the statistical analysis to compare the groups. The average live weight and average daily gain were calculated. Daily inspection of the poultry stock was carried out with recording of cases of mortality. Feed consumption was recorded for each group to determine the feed cost per 1 kg of gain. Individual weightings of broiler chickens were carried out at 0 and 7 days using Mercury 122ACFJR-600.01 electronic laboratory scales (Mertech, Shchyolkovo, Russia) with a resolution of 0.01 g and at other stages of ontogenesis using M-ER 223 AC-15.2 Mary LCD electronic scales (Mertech, Shchyolkovo, Russia) with a resolution of 2 g. Live weight was recorded in special forms. Based on the average values for the group, the average daily gain was calculated using the following formula [21]:

(2) $$ADG=\frac{W2-W1}{t2-t1}$$

where W2—the live weight of broilers at the end of the growing period (g); W1—the live weight of broilers at the beginning of the growing period (g); t2—the age of chickens at the end of the growing period (days); and t1—the age of chickens at the beginning of the growing period (days).

Based on the results of monitoring feed consumption and determining the gross gain in live weight of chickens in each group, feed costs per 1 kg of gain were calculated using the following formula [21]:

(3) $$FC=\frac{individual\;feed\;intake\;during\;the\;rearing\;period,g\;}{average\;absolute\;growth\;of\;chickens,\;g}$$

where the individual feed intake during the rearing period was obtained by dividing the gross feed intake of the group by the number of chickens and taking into account changes in the chickens’ livability during the experiment.

The preservation of poultry stock was calculated based on the percentage ratio of the number of individuals at the end of the rearing period to the stock at the beginning of the experimental period in each group, in accordance with the results of accounting for chicken mortality.

Taking into account the calculated growth indicators for broiler chickens, the European efficiency index (EEI) was determined using the following formula [21]:

(4) $$EEI=\frac{Survivability,\%\times Live\;weight\;of\;1\;head,kg}{Age\;of\;slaughter,days\times Feed\;costs\;per\;1\;kg\;of\;gain,kg}\times 100$$

The anatomical dissection of broiler carcasses was performed in a specially equipped room using surgical instruments. The results were recorded. Internal organs, legs, breasts, other muscles, skin, bones, and fat were weighed, and the following meat productivity indicators were calculated: the weight of the eviscerated carcass (g), dressing yield (%), and yield of muscles and fat (%). Mercury 122ACFJR-600.01 laboratory scales (Mertech, Shchyolkovo, Russia) with a sensitivity of 0.01 g were used for weighing.

### 2.4. Sample Collection

At the ages of 22 and 36 days, 3 cockerels with average live weight were selected from each group (a total of 9 cockerels aged 22 days and 9 cockerels aged 36 days). Blood was collected from the subcutaneous vein (*Cutanea ulnaris*) on the inner side of the wing above the elbow joint into Lab-Vac tubes (Shandong Chengwu Medical Products Factory, Chengwu County, Shandong Province, China) with a coagulation activator in a volume of 2–3 mL. Subsequently, the serum was separated using a Mikro 220 centrifuge (Hettich North America, Beverly, MA, USA) and sent for laboratory analysis.

In accordance with the Recommendations of the Collegium of the Eurasian Economic Commission “On Guidelines for Working with Laboratory (Experimental) Animals in Preclinical (Non-Clinical) Research”, 3 cockerels from each group aged 22 days were euthanized (a total of 9 cockerels from which blood was previously drawn). Subsequently, autopsies were conducted, and samples of breast muscle tissues, cecal diverticula, and ileum were taken under sterile conditions. The samples were stabilized using IntactRNA fixative (Evrogen, Moscow, Russia) and stored at −20 °C until laboratory analyses were conducted. The number of individuals selected for molecular genetic studies was based on the results of existing research [22].

### 2.5. Biochemical Analysis of Blood

Biochemical studies of blood serum were carried out at the Department of Physiology and Biochemistry of Farm Animals of the All-Russian Institute of Animal Husbandry named after L.K. Ernst (Podolsk, Russia) using an automatic biochemical analyzer ChemWell (Awareness Technology, Palm City, FL, USA) with reagents from Analyticon Biotechnologies AG (Lichtenfels, Germany), Spinreact S.A. (Girona, Spain), and JSC Diakon (Pushchino, Russia) according to the methods recommended by the manufacturers of the equipment and reagents. Antioxidant protection indicators in blood serum were determined by the concentration of active products that react with thiobarbituric acid (TBA-AP) using kits from Agat-Med LLC (Balashikha, Russia), while ceruloplasmin (CP) activity was determined using the Revin method [23]. The total concentration of water-soluble antioxidants (TSWA) was determined by the amperometric method on a Tsvet Yauza-01-AA device with a detector (Scientific-Production Association Khimavtomatika, Moscow, Russia). The ratio of TBA-AP to CP was determined by calculation.

### 2.6. RNA Isolation and Real-Time PCR

The studies of gene transcriptional activity were carried out at the shared-use educational and scientific center “Service Laboratory for Complex Analysis of Chemical Compounds” of the Russian State Agrarian University—Moscow Agricultural Academy named after K.A. Timiryazev (Moscow, Russia). Total RNA was isolated from the samples using the RNA Solo kit (Eurogen, Moscow, Russia), after preliminary homogenization and lysis of the tissues using a TissueLyser II device (Qiagen, Venlo, The Netherlands). cDNA was synthesized on the matrix of the isolated RNA using reverse transcriptase from the Magnus kit (Eurogen, Moscow, Russia) on a C1000 Touch device (Bio-Rad, Berkeley, CA, USA). The quality and quantity of the isolated RNA and synthesized cDNA were analyzed on a Fluo-200 device (Allsheng, Hangzhou, China) using the QuDu ssDNA kit (Thermo Fisher Scientific, Waltham, MA, USA). All RNA extraction and cDNA synthesis were performed according to the molecular assay kit manufacturers’ instructions. Synthesized cDNA was stored at −30 °C.

For amplification of the selected genes, gene-specific primer sequences (Table 3) were used. They were selected based on the results of a review of the world scientific literature and verified using the Ensembl genome browser. The real-time polymerase chain reaction (RT-PCR) was measured using a CFX96 Touch amplifier (Bio-Rad, Berkeley, CA, USA) and a 5X qPCRmix-HS SYBR kit (Eurogen, Moscow, Russia). The housekeeping gene encoding the β-actin protein ACTB was used as a control gene. The amplification mode was as follows: 3 min at 95 °C (preliminary denaturation), 30 s at 95 °C, 30 s at 60 °C, and 30 s at 72 °C (40 cycles). The relative expression level was assessed using the 2^−ΔΔCT^ method [24].

### 2.7. Statistical Analysis

The data was analyzed using single-factor analysis of variance (ANOVA) using the GLM procedure at SPSS 23 (SPSS Inc., Chicago, IL, USA, 2023) after the Shapiro–Wilk test was used to verify the normality. The use of statistical analysis employing orthogonal polynomial contrasts was justified by evaluating the effects of different saponin concentrations in quinoa grain (0.24% in the SAP group, 5.20% in the SAP-FREE group). The Tukey criterion was used to compare differences between the averages with a probability of 5% regarding growth, meat productivity, and biochemical blood parameters of the experimental groups. The results are presented as averages and cumulative standard error of the mean (SEM), and the significance level was set at *p* ≤ 0.05. To compare gene expression of the experimental groups against the control, the Student’s *t*-test was used for ΔCt values. A difference was considered significant at *p* ≤ 0.05. A mathematical model was used in the statistical analysis:

Xijk = μ + Ai + eijk

where Xik = an individual observation; μ = overall mean; Ai = effect of the ith treatment; and eik = random error.

## 3. Results

### 3.1. Growth and Meat Productivity of Broiler Chickens

According to the results of the analysis of the dynamics of live weight of broiler chickens, no reliable differences were found between the experimental groups. However, the final weighing showed the live weights of the SAP group chickens were closest to the CON group values (without separation by sex—0.9% lower, cockerels—3.3% higher, hens—0.8% lower), while the SAP-FREE group chickens were inferior to the control (without separation by sex—2.6% lower, cockerels—0.6% lower, hens—4.9% lower) (Table 4). The results of growing broiler chickens showed no negative impact of using quinoa grain containing saponins in broiler chicken feed on the level of poultry productivity. The values of average daily gain, poultry stock survival, and the European productivity index in the SAP group were at the same level as the control, while the SAP-FREE group was characterized by a relatively high mortality (10%) and the lowest value of the European productivity index.

The anatomical cutting revealed some changes in the anatomical structure of broiler chickens and, accordingly, in their meat productivity (Table 5). A significant difference was found only for cockerels. In particular, the SAP group demonstrated the best development of the thigh muscles—9.6% higher than in the control (*p* = 0.008). The experimental group chickens were characterized by the lowest yield of shin compared to the control—1 abs.% (*p* = 0.003) in the SAP group and 0.7 abs.% (*p* = 0.014) in the SAP-FREE group. The highest yield of other muscles was noted in chickens fed diets supplemented with quinoa containing saponins. The value of this indicator in the SAP group was 2.9 abs.% higher than in the SAP-FREE group (*p* = 0.007). It is important to note a significant decrease in the weight and fat yield in the carcasses of the cockerels of the SAP-FREE experimental group. The fat weight in the carcasses of chickens of this group was 56.4% (*p* = 0.019) lower than in the CON group and 62.0% (*p* = 0.007) lower than in the SAP group, while the fat yield was lower by 1.7 (*p* = 0.015) and 2.0 abs.% (*p* = 0.008), respectively. Despite the absence of a reliable difference, the cockerels of the SAP group in terms of slaughter yield and muscle yield from live weight were at the same level as the control, while the representatives of the SAP-FREE group were inferior to the control in these indicators by 3.1 and 1.1 abs.%, respectively. The hens of this group surpassed the control in slaughter yield but were at the same level in muscle mass yield. It should also be noted that the highest values of the ratio of edible parts to inedible ones were in the SAP group, both in cockerel carcasses and in hens.

### 3.2. Biochemical Blood Parameters of Broiler Chickens

Table 6 shows the biochemical analysis of the blood of broiler chickens for two age periods. No changes were observed at 22 days of age, but some changes in the biochemical composition of the blood serum were found at 36 days of age, when quinoa was eliminated from the chicken diet (Table 6). In particular, AST activity in the SAP-FREE group was 34.0% (*p* = 0.019) higher than in the CON group and 36.2% (*p* = 0.016) higher than in the SAP group. Creatinine blood concentration in SAP-FREE group chickens was 15.7% higher compared to the control (*p* = 0.008).

### 3.3. Expression of Genes of Broiler Chickens

**Figure 2:**
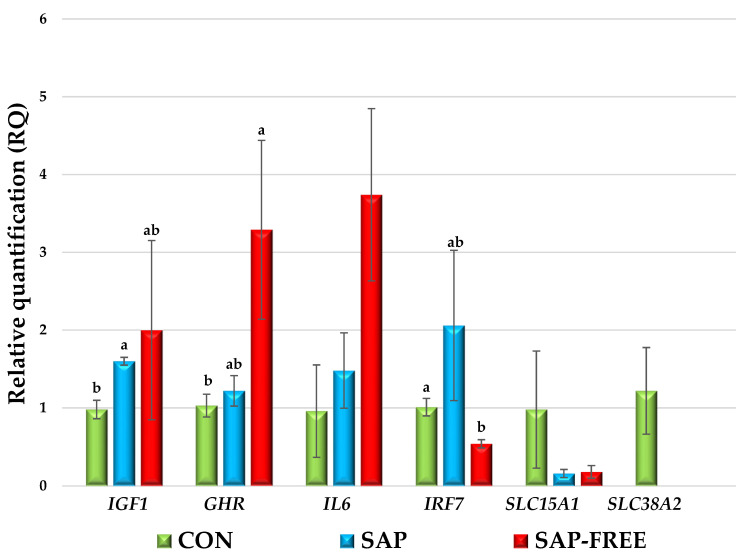
Relative quantification (RQ) of expression of genes in the pectoral muscles, jejunum, and cecum of broiler chickens (results are presented as the mean of the standard error of the mean (M ± SEM) for mRNA expression; a,b—difference in ΔCt values is statistically significant at *p* ≤ 0.05 according to the t-criterion).

**Figure 3:**
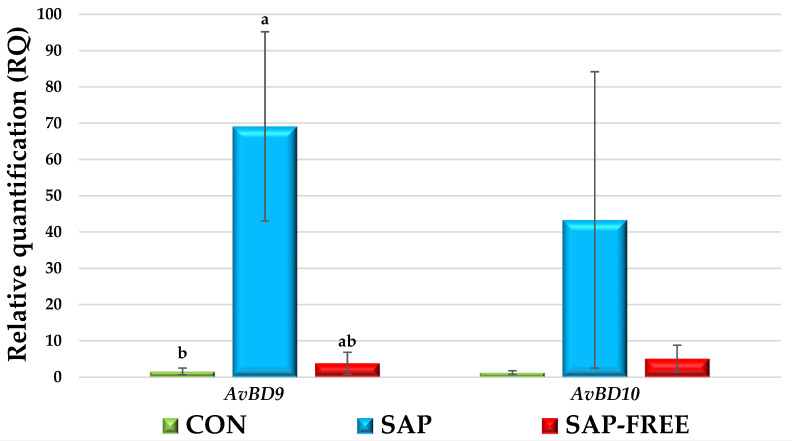
Relative quantification (RQ) of expression of genes *AvBD9-10* in cecum of broiler chickens (results are presented as the mean of the standard error of the mean (M ± SEM) for mRNA expression; a,b—difference in ΔCt values is statistically significant at *p* ≤ 0.05 according to the t-criterion).

Figure 2 and Figure 3 show the expression values of genes associated with growth, immunity, and nutrient transport. The results of molecular genetic studies indicated the transcriptional activity of the *IGF1* (insulin-like growth factor 1) gene increased by 1.6 times (*p* = 0.014) in the tissues of the pectoral muscle of the SAP group chickens, while the expression of the *AvBD9* (avian beta-defensin 9) gene significantly increased by 69.1 times (*p* = 0.010) in the tissues of the caecum. In the SAP-FREE group, the expression of the GHR (growth hormone receptor) gene increased by 3.3 times (*p* = 0.039) in the tissues of the pectoral muscle, while the expression of the *IRF7* (interferon regulatory factor 7) gene decreased to a value of 2^−ΔΔCT^ 0.54 (*p* = 0.017) in the tissues of the caecum. It should be noted that despite the absence of reliable differences, the use of quinoa grain with both high and low saponin content reduced the activity of the *SLC15A1* gene (solute carrier family 15 member 1) to the same extent to values of 2^−ΔΔCT^ 0.16–0.18 and also probably led to the blocking of transcriptional processes relative to the *SLC38A2* gene (solute carrier family 38 member 2), since the cDNA of this gene was not detected according to the results of RT-PCR.

## 4. Discussion

### 4.1. Growth and Meat Productivity of Broiler Chickens

The lack of significant differences in the growth and some performance indicators of broiler chickens when fed different variants of quinoa grain compared to the control can likely be explained by the fact that the nutritional and chemical composition of the compound feeds in the experimental groups was identical. Overall, a number of studies have indicated no substantial changes in the productivity levels of the poultry when incorporating unconventional sources of energy and protein into their diets, provided that the nutritive value of the feed is maintained. In the work of Danek-Majewska et al., which investigated the partial replacement of soybean meal with chickpea grain (*Cicer arietinum* L.) in compound feed for broiler chickens, no reliable difference was noted in the values of productive indicators such as live weight at the end of rearing, average daily gain, and feed conversion [30]. However, the results of control weighing and calculation of the productivity index indicated some advantage of chickens fed quinoa grain with saponins. One possible explanation for this result may be the difference in the nature of assimilation of nutrients of different quinoa grains by broiler chickens. Previously, Nasir et al. conducted a comparative assessment of the biochemical and functional properties and digestibility of proteins of quinoa grain of four different genotypes in vivo in rats and in vitro. They were able to establish that quinoa grain of different genotypes was characterized by different values of protein efficiency coefficient—from 3.45 to 3.78, protein ratio—from 3.90 to 4.69, protein utilization—from 70.75% to 73.78%, true protein digestibility—from 87.66% to 90.57%, biological value of protein—from 79.15% to 81.74%, and in vitro digestibility—from 75.95% to 78.11% [31].

The survival of poultry stock in our study indicated better viability of individuals consuming quinoa grain with saponins, whereas feeding quinoa free of saponins, on the contrary, was accompanied by a relatively high level of mortality. This is partly explained by the results of molecular genetic studies of gene expression, which showed an increased transcriptional activity of the pro-inflammatory gene IL6, decreased expression of the antiviral gene IRF7, and significantly lower expression of antimicrobial peptides in the caecum tissues of chickens in the SAP-FREE group compared to the SAP group. These changes may be associated with impaired intestinal health and reduced overall resistance of the birds. Thus, we assume that saponins contribute to maintaining intestinal health and the overall immune resilience of the birds.

The lowest feed costs per 1 kg of gain in the SAP-FREE group compared to the CON and SAP groups are due to lower feed intake, apparently related to the feeling of satiety in chickens fed quinoa grain that does not contain saponins. Probably, the absence of saponins reduces irritation of the digestive system and contributes to a more comfortable feeding experience, which affects the birds’ feeling of satiety. Earlier studies on broiler chickens have already shown a reduction in feed intake when quinoa is used in the diet [12]. It has also been previously established that when rats are fed diets that differ in protein source, their feed intake level and blood satiety hormone concentrations change. Thus, over 15 days of the experiment, rats consumed 22% less feed with quinoa grain than rats fed a casein diet, which is equivalent to a 13% reduction in dietary energy intake. At the same time, after feeding, the level of ghrelin, leptin, and cholecystokinin in the blood serum of these rats decreased [32]. A similar pattern was found in the studies by Noratto et al. They showed that the use of a diet containing quinoa flour, similar in nutritional value to the control, contributed to the smallest gains in live weight of mice in the first 5 weeks of the experiment due to the lowest feed consumption. In experimental individuals, the level of total cholesterol also decreased, and the level of insulin in the blood serum increased [33]. Based on the studies conducted, the authors suggest the possibility of using quinoa for the prevention of obesity. To understand the reasons for changes in feed consumption by chickens when quinoa grain is included in their diet, it would be advisable to study the concentration of hormones related to satiety in the birds’ blood in future research.

This is confirmed to some extent by the results of our studies. Quinoa that does not contain saponins had a certain effect on the nature of adipose tissue formation. Based on the results of anatomical cutting of broiler chickens, it was found that the mass and yield of abdominal and subcutaneous fat in the carcasses of cockerels of the SAP-FREE group were significantly reduced in comparison with the CON and SAP groups. Quinoa is rich in fiber, protein, and polyphenols [34], which have functional properties that help improve metabolism, reduce fat mass, and protect against metabolic disorders associated with obesity. The difference with the SAP group is likely due to saponins binding to cholesterol and reducing its activity and regulating the expression of key enzymes or proteins involved in cholesterol synthesis and metabolism [35]. This can limit metabolic efficiency, slow down lipolysis (fat breakdown), and prevent the fat reduction effect from manifesting. Elbaz et al. also found a statistically significant decrease in the proportion of abdominal fat per total carcass weight, from 2.08% for control group chickens to 1.72–1.81% (*p* = 0.02) in chickens fed a diet where 5, 10, and 15% of the total feed weight was quinoa grains [13]. Based on a review of numerous medical studies, Little et al. look at quinoa as a functional food component that provides potential protection against metabolic complications associated with obesity and type 2 diabetes due to substances such as fiber, protein, 20-hydroxyethyl carotene, and polyphenols [36]. Thus, the results of the present study are consistent with this concept and indicate the possibility of further studying the use of saponin-free quinoa as a functional feed in animal nutrition for the prevention of obesity (breeding livestock) or in the production of dietary meat. It should be noted that a decrease in the fat content was found only in the carcasses of cockerels. The same pattern was found by Naımatı et al. when using quinoa seed extract in the diet of Japanese quail. In their study, the proportion of abdominal fat in quail carcasses decreased in males from 2.27 to 1.67–1.98% when using the extract in an amount of 0.1–0.4 g/kg, while in females, on the contrary, it increased from 1.56 to 1.97–2.22%; however, the changes were not reliable (*p* = 0.261, *p* = 0.223) [37]. In general, this may be explained by the difference in the hormonal system and biological response of males and females to the action of alimentary factors. Previously, we established a similar pattern when using various phytobiotics in the diet of broiler chickens, with hens (*p* ≤ 0.01) differing significantly from cockerels (*p* ≤ 0.05) in terms of fat deposition [21].

In addition to changes in the amount of abdominal fat in carcasses, a decrease in the yield of shin was found in groups of chickens fed compound feed with quinoa. However, the reduction in shin yield relative to the total carcass weight was compensated by an increase in the yield of breast muscles, which are of greater interest from a consumer perspective. The SAP group also showed the best development of the thigh muscles, as well as the muscles of the back, wings, and neck, compared to the SAP-FREE group. We assume that saponins may have a selective effect on metabolism and blood supply, leading to more active development of muscles involved in movement and posture maintenance, such as the muscles of the back, thighs, and wings. In the study by Naımatı et al., it was also noted that significant changes in the composition of quail carcasses when using quinoa extract were characteristic only of the weight of the wing muscles, which indicates a similar effect of this feed on muscle development and confirms the reliability of the results [37]. However, significant differences in other parameters of meat productivity were not found when using quinoa grain. In the above-mentioned study of Elbaz et al., no statistically significant differences were found in the yield of breast and thigh muscles when including quinoa grain in the compound feed, regardless of the level of addition. The study showed that the slaughter yield increased only when quinoa was included in the diet in the amount of 10–15%, and the difference was not significant with a proportion of quinoa in the diet of 5%, as in our study [13]. In general, broiler chickens consuming quinoa with both high and low saponin content were at the same level in terms of meat productivity and did not differ significantly from the control group.

Probably, the absence of quinoa exclusion from the diet 5 days before slaughter could have differently affected the results of control weighing and carcass quality. However, this procedure was introduced to avoid deterioration of the commercial qualities of the carcasses. Further studies are planned to investigate the organoleptic properties of poultry meat with varying durations of quinoa grain feeding.

### 4.2. Biochemical Blood Parameters of Broiler Chickens

First of all, it was established that during the period of feeding broiler chickens with quinoa grains as a component of compound feed, there were no significant changes in the biochemical composition of blood serum and antioxidant protection indices. This indicates the absence of a negative effect of using this crop in poultry nutrition on their metabolism.

At the end of poultry rearing, when quinoa grains were excluded from the diet, some changes in the biochemical composition of blood serum were observed in the SAP-FREE group. In this case, significantly increased AST activity and creatinine concentration are an indicator of a sharp intensification of protein metabolism, inhibited during the use of quinoa in the diet. This assumption is consistent with the description of live weight gain and feed consumption, which probably indicates low availability of saponin-free quinoa protein compounds for the chickens. In the SAP group, low protein bioavailability was most likely compensated by the beneficial effect of saponins. This is partly supported by the study by Son et al., where a slight tendency towards a decrease in AST activity and creatinine levels was noted in the serum when crude protein in the diet of broiler chickens was reduced. However, the differences in this experiment were not as significant as in our study [38]. Horio et al. also noted that AST activity increased significantly in rats fed a high-protein diet [39].

It should be noted that despite differing fat deposition in the carcasses of chickens from the control and experimental groups, no differences were found in the parameters of lipid metabolism in blood serum. We assume that this is related to the function and activity of the relevant enzymes and hormones that maintain homeostasis, despite variations in lipid accumulation in adipose tissue.

### 4.3. Expression of Genes of Broiler Chickens

*IGF1* plays an important role in the processes of cell growth, differentiation, and apoptosis [40]. The physiological function of this gene and its product, which ensures the growth of skeletal muscles, allows *IGF1* to be considered one of the most promising candidate genes for assessing meat productivity traits in chickens [41,42]. The growth hormone receptor (*GHR*) activates several signaling pathways under the influence of growth hormone and is also involved in the regulation of skeletal muscle growth [43].

Jia et al. found that *IGF1* gene expression in the pectoral muscles did not significantly correlate with live weight and average daily gain in broiler chickens, while expression of this gene in the liver and thigh muscles positively correlated with the level of gain. Regarding the *GHR* gene, Jia et al. reported a significant inverse relationship between its expression in pectoral muscle tissues and average daily gain (r = −0.317, *p* ≤ 0.05) [44]. This explains our results. In the SAP group, an increase in *IGF1* gene expression was not accompanied by significant changes in the live weight of chickens, while an increase in the transcriptional activity of the *GHR* gene in the SAP-FREE group actually corresponded to the lowest value of average daily gain.

It is known that dwarf chickens, in which normal protein function is impaired due to exonic mutations in the *GHR* gene, have higher fat deposition. Zhao et al. found that *GHR* is involved in lipid metabolism, suppressing adipogenic differentiation of bone marrow mesenchymal stem cells, lipid peroxidation, and fat deposition in vivo and in vitro [45,46]. The results of our studies are consistent with these statements: in the SAP-FREE group, which was characterized by high *GHR* expression, a decrease in the weight and fat yield of cockerel carcasses was found.

Type I interferons (IFNs) in mammals are considered to be key activators of a signaling pathway involving more than 300 genes in response to viral infections, making them key elements of innate immunity against viruses. In chickens, as in mammals, a strong induction of type I interferons is observed in response to infection with various viruses, such as Newcastle disease virus (NDV) or AIV [47]. It was established that in chickens, *IRF7* can modulate a wide range of cellular processes in the host innate immune response to viral infection [48]. Thus, the decrease in *IRF7* expression in the SAP-FREE group may be an indicator of deterioration of innate immunity and increased susceptibility to viruses. In the study by Laptev et al., infection with Salmonella Enteritidis led to immunosuppression on day 23 post-infection, which was confirmed, among other things, by a tendency of decreased *IRF7* expression [22]. The decrease in the activity of this gene in the SAP-FREE group may also be associated with a decrease in the resistance of broiler chickens and explains the high mortality rate in the group. In the SAP group, no such changes were observed because quinoa saponins, such as phytolaccagenic, oleanolic, and serjanic acids; hederagenin, 3β,23,30 trihydroxy olean-12-en-28-oic acid; 3β-hydroxy-27-oxo-olean-12en-28-oic acid; and 3β,23,30 trihydroxy olean-12-en-28-oic acid, exhibit anti-inflammatory activity. In the SAP-FREE group, the absence of these compounds caused a negative effect, apparently associated with other quinoa constituents, likely polysaccharides, which can also influence the nature of immune processes [49].

A multiple increase in the expression of the avian beta-defensin 9 (*AvBD9*) gene was found in the caecum of the SAP chickens. Avian β-defensins (*AvBD*) are genes that encode antimicrobial peptides that neutralize pathogenic bacteria, fungi, and viruses. Together with cytokines and toll-like receptors, β-defensins are key biomarkers of innate immunity [50,51]. Their functions include many immunomodulatory properties, including chemotaxis, activation of immune cells and mucin synthesis, modulation of inflammation and autophagy, and playing a key function is antibacterial activity [52]. The significant increase in *AvBD9* expression under the influence of quinoa with saponins probably indicates a beneficial effect of saponins on the health of the poultry intestine and is confirmed by the highest survival rate in the SAP group, proving the immunostimulating properties of saponins. This is related to the structure of saponins. Glycosides consisting of a hydrophobic aglycone (steroidal or triterpenoid) and hydrophilic carbohydrate [11] chains are capable of binding to the cell membranes of intestinal epithelial cells. It is likely that saponins alter the physiological processes occurring in intestinal cells, including transcription and translation. Note that the expression of *AvBD9* in the experimental groups changed insignificantly but with the same pattern as *AvBD9*. Previously, we noted the same trend under the influence of other nutritional factors [21].

Peptide transporter 1 *PepT1* (*SLC15A1*) transports feed amino acids into intestinal epithelial cells in the form of di- and tripeptides [53]. This gene and the product of its expression play a significant role in protein nutrition processes and facilitate the absorption of a substantial portion of exogenous proteins of animal, plant, and microbiological origin from the diet, as well as endogenous proteins of epithelial and microbiological origin, which are synthesized in the intestinal lumen and broken down by digestive and/or microbial enzymes [54]. In our study, *SLC15A1* expression in both experimental groups significantly decreased, although the decrease was not significant, while *SLC38A2* expression was not detected at all in the experimental groups. This confirms our assumptions that broiler chickens use dietary protein less efficiently due to the inclusion of quinoa grain in the diet and also most likely indicates the presence of protease inhibitors in quinoa that exhibit high activity. The presence of protease inhibitors in quinoa has been described in some studies [8,55]. Due to the presence of antinutritional factors, proteins in the intestine are not completely broken down into peptides and amino acids, which reduces the availability of nutrients for absorption. As a result, the activity and expression of peptide transporters (such as *SLC15A1/SLC38A2*), responsible for transporting amino acids in the form of di- and tripeptides, decrease because the body requires less transport of these nutrients. Thus, in the future, the possibility of including quinoa grain with additional enzymatic treatment in the diet of broiler chickens should be further studied. Low protein bioavailability in the SAP group was compensated by the beneficial effect of saponins, due to which individuals of this group were more similar to individuals of the control group in terms of productivity. Together, this indicates the feasibility of using quinoa grain without preliminary economic costs for processing from saponins.

In conclusion, it should be noted that partly replacing corn, the most traditional source of energy in poultry diets, with quinoa grain does not lead to a decrease in the productivity of broiler chickens, which indicates some advantages of this strategy for feeding broilers. Climate change, including an increase in CO_2_ in the atmosphere, a significant increase in temperature, and a change in the precipitation regime, leads to a significant decrease in moisture supply to agricultural crops. In this regard, the use of crops with low water consumption and high yields is becoming increasingly widespread. While corn is susceptible to temperature stress and drought, crops (such as sorghum, millet, and quinoa) adapted to conditions with limited access to irrigation water and resistant to climatic risks can replace corn in areas with limited water supply, including the central part of Russia [56,57]. Recently, quinoa has increasingly been referred to as a “new generation crop” [58], as it can be grown in areas with irregular rainfall, expected water shortages, and other extreme conditions [34,59]. In 2024, the global quinoa market reached USD 1.4 billion. By 2033, according to IMARC Group forecasts, the market will reach USD 2.5 billion with a projected average annual growth rate of 6.75% from 2025 to 2033 [60]. Finally, it should be noted that quinoa is relatively cheap compared to corn, more than two times cheaper than average prices on the Russian Federation market in 2025.Therefore, partly replacing corn (from 3 to 5% of the feed weight) with quinoa grain in feed for broiler chickens does not reduce the productivity of poultry but reduces the cost of poultry meat production and allows the use of more affordable plant materials.

## 5. Conclusions

Comparing the efficiency of using quinoa grain with and without pre-treatment to remove saponins, the latter option shows certain advantages. Therefore, it is recommended that further studies of quinoa as a feed crop in poultry farming focus on quinoa grain containing saponins without pre-treatment, thus avoiding additional costs for feed preparation. However, it is advisable to include additional enzymatic treatment with proteases to reduce antinutritional factors and to also investigate the effect of quinoa grains on a larger sample and with a longer duration of feeding.

## Figures and Tables

**Table 1:** Characteristics of the compound feeds used.

Component of Compound Feeds (%)	Compound Feed Without Quinoa Grain			Compound Feed with Quinoa Grain		
	0–10 Starter	11–22 Grower	23–35 Finisher	0–10 Starter	11–22 Grower	23–35 Finisher
Composition of compound feeds						
Wheat	52.70	36.89	38.63	53.87	38.10	40.21
Corn	9.00	20.00	22.40	5.77	15.06	16.95
Quinoa	-	-	-	3.00	5.00	5.00
Full-fat soybeans	11.00	10.00	15.00	11.06	10.00	14.95
Soybean meal	4.80	10.34	3.00	4.53	9.56	1.91
Sunflower meal	9.00	10.00	9.55	8.63	10.00	10.00
Fish meal	8.50	3.20	3.00	8.50	3.20	3.00
Sunflower oil	1.89	5.00	4.00	1.59	4.56	3.63
Lysine monohydrochloride 98%	0.30	0.20	0.25	0.30	0.20	0.26
DL-methionine 98.5%	0.20	0.20	0.20	0.21	0.21	0.21
L-threonine 98%	0.06	0.21	0.21	0.06	0.21	0.21
Table salt	0.10	0.16	0.16	0.10	0.16	0.16
Monocalcium phosphate	0.60	1.00	1.00	0.55	0.96	0.93
Limestone flour	1.10	1.50	1.30	1.08	1.48	1.28
Sodium sulfate	0.20	0.25	0.25	0.20	0.25	0.25
Vitamin B4 80%	0.05	0.05	0.05	0.05	0.05	0.05
Premix	0.50	1.00	1.00	0.50	1.00	1.00
Nutritional value and chemical composition of compound feeds						
Metabolic energy (kcal/100 g)	296.25	310.99	312.35	296.24	310.98	312.39
Crude protein (%)	22.08	20.76	19.05	22.08	20.76	19.05
Crude fiber (%)	3.80	3.88	3.94	3.78	3.92	4.04
Calcium (%)	0.99	0.96	0.86	0.99	0.96	0.86
Available phosphorus (%)	0.45	0.41	0.40	0.45	0.41	0.40
Total lysine (%)	1.39	1.19	1.11	1.39	1.19	1.11
Total methionine + cystine (%)	0.93	0.88	0.83	0.93	0.88	0.83

**Table 2:** Amino acid composition of quinoa grain.

Amino Acid Content (g/100 g Protein)	Value
Valine	3.42
Leucine + isoleucine	8.22
Lysine	4.26
Methionine + cystine	0.84
Threonine	3.86
Phenylalanine	3.11
Total protein (%)	13.36

**Table 3:** Nucleotide sequence of primers.

Gene	Primers	Author
The housekeeping gene		
*ACTB* (β-actin)	F: CTGTGCCCATCTATGAAGGCTA R: ATTTCTCTCTCGGCTGTGGTG	Laptev G.Yu. et al., 2023 [25]
Genes associated with growth and development of skeletal muscles (pectoral muscle tissue)		
*IGF1* (insulin-like growth factor 1)	F: GCTGCCGGCCCAGAA R: ACGAACTGAAGAGCATCAACCA	Tyurina D.G. et al., 2022 [26]
*GHR* (growth hormone receptor)	F: AACACAGATACCCAACAGCC R: AGAAGTCAGTGTTTGTCAGGG	Kirrella A.A. et al., 2021 [27]
Immunity-related genes (cecal tissue)		
*AvBD9* (avian beta-defensin 9)	F: AACACCGTCAGGCATCTTCACA R: CGTCTTCTTGGCTGTAAGCTGGA	Laptev G.Yu. et al., 2023 [25]
*AvBD10* (avian beta-defensin 10)	F: GCTCTTCGCTGTTCTCCTCT R: CCAGAGATGGTGAAGGTG	
*IL6* (interleukin 6)	F: AGGACGAGATGTGCAAGAAGTTC R: TTGGGCAGGTTGAGGTTGTT	Tyurina D.G. et al., 2022 [26]
*IRF7* (interferon regulatory factor 7)	F: ATCCCTTGGAAGCACAACGCC R: CTGAGGCAACCGCGTAGACCTT	Laptev G.Yu. et al., 2023 [25]
Genes associated with nutrient transport (jejunum tissue)		
*SLC15A1* (solute carrier family 15 member 1)	F: AATTGGGCAGGCAGTCATGG R: AGCGCGATGAGAATCAAGCC	Park J.H. et al., 2020 [28]
*SLC38A2* (solute carrier family 38 member 2)	F: CGCAGGACACTGGTATCTTAAT R: GCCACTGGTATAGCCCAAATA	Fagundes N.S. et al., 2020 [29]

**Table 4:** Chicken broiler productivity indicators.

Age/Sex	Groups			*p*-Value		
	CON	SAP	SAP-FREE	Anova	Linear	Quadratic
Dynamics of live weight of broiler chickens (g) (*n* = 50)						
0 days	45.7 ± 0.49	45.5 ± 0.49	45.6 ± 0.49	0.958	0.970	0.770
7 days	204.6 ± 2.10	202.6 ± 2.10	200.3 ± 2.17	0.365	0.157	0.954
14 days	511.8 ± 6.72	495.9 ± 6.72	498.7 ± 6.87	0.209	0.178	0.258
21 days	1035.2 ± 13.17	1014.3 ± 13.17	1004.2 ± 13.32	0.244	0.101	0.737
28 days	1655.1 ± 25.86	1610.9 ± 25.54	1600.5 ± 26.55	0.293	0.144	0.593
36 days	2162.9 ± 45.11	2142.9 ± 44.48	2107.0 ± 47.94	0.694	0.398	0.886
Cockerels	2260.6 ± 52.72	2336.0 ± 59.78	2246.4 ± 55.92	0.509	0.854	0.252
Hens	2059.5 ± 56.48	2042.9 ± 50.82	1958.4 ± 60.13	0.429	0.226	0.607
Calculated indicators						
Absolute gain (g)	2117.2	2097.4	2061.4	n/a		
Average daily gain (g)	58.8	58.3	57.3	n/a		
Chickens survival (%)	96.0	98.0	90.0	n/a		
Feed-to-gain ratio (kg/kg)	1.86	1.90	1.84	n/a		
European productivity index (units)	310.10	307.02	286.28	n/a		

Values are expressed as LS means ± standard error; n/a—not applicable, since the values are calculated.

**Table 5:** The meat productivity of broiler chickens.

Indicators	Groups			SEM	*p*-Value		
	CON	SAP	SAP-FREE		Anova	Linear	Quadratic
Cockerels, *n* = 3							
Gutted carcass weight (g)	1622.3	1690.7	1615.2	47.28	0.501	0.919	0.260
Slaughter yield (%)	74.0	73.6	70.8	1.84	0.458	0.267	0.610
Pectoral muscle weight (g)	398.0	430.1	443.9	18.55	0.275	0.131	0.701
Pectoral muscle yield (%)	18.2	18.7	19.4	0.78	0.537	0.286	0.925
Thigh muscle weight (g)	211.5 ^b^	231.7 ^a^	218.4 ^ab^	3.70	0.022	0.235	0.010
Thigh muscle yield (%)	9.7	10.1	9.6	0.16	0.122	0.756	0.050
Shin weight (g)	152.8	138.0	143.7	4.22	0.117	0.177	0.094
Shin yield (%)	7.0 ^a^	6.0 ^b^	6.3 ^b^	0.14	0.007	0.014	0.010
Other muscle weight (g)	143.2 ^ab^	176.1 ^a^	110.4 ^b^	10.71	0.014	0.074	0.009
Other muscle yield (%)	6.5 ^ab^	7.7 ^a^	4.8 ^b^	0.49	0.019	0.050	0.017
Total skeletal muscle weight (g)	905.6	975.9	916.5	28.60	0.252	0.797	0.113
Muscle yield from live weight (%)	41.3	42.4	40.2	1.18	0.443	0.513	0.284
Fat weight (g)	63.6 ^a^	72.9 ^a^	27.7 ^b^	7.99	0.016	0.019	0.032
Fat yield (%)	2.9 ^a^	3.2 ^a^	1.2 ^b^	0.35	0.016	0.015	0.043
Liver weight (g)	56.9	60.6	56.1	3.65	0.660	0.882	0.388
Heart weight (g)	12.0	14.5	15.7	1.38	0.222	0.101	0.704
Gizzard weight without cuticle (g)	17.0	19.9	19.0	1.00	0.197	0.219	0.170
Ratio of edible parts to inedible parts	2.0	2.0	1.8	0.19	0.622	0.403	0.657
Hens, *n* = 3							
Gutted carcass weight (g)	1469.5	1399.0	1467.8	26.26	0.177	0.965	0.074
Slaughter yield (%)	73.3	69.9	74.2	1.05	0.061	0.554	0.025
Pectoral muscle weight (g)	355.1	364.0	384.9	13.43	0.340	0.168	0.728
Pectoral muscle yield (%)	17.7	18.2	19.5	0.77	0.329	0.164	0.691
Thigh muscle weight (g)	167.4	253.7	158.9	25.74	0.074	0.823	0.028
Thigh muscle yield (%)	8.3	12.7	8.0	1.24	0.069	0.865	0.026
Shin weight (g)	117.6	91.2	131.2	14.50	0.220	0.533	0.111
Shin yield (%)	5.9	4.6	6.6	0.75	0.224	0.503	0.116
Other muscle weight (g)	165.0	108.8	128.5	15.09	0.095	0.139	0.086
Other muscle yield (%)	8.2	5.4	6.5	0.79	0.111	0.168	0.092
Total skeletal muscle weight (g)	805.1	817.7	803.5	24.02	0.902	0.965	0.665
Muscle yield from live weight (%)	40.2	40.9	40.6	1.34	0.938	0.828	0.789
Fat weight (g)	40.1	55.7	53.3	8.33	0.418	0.306	0.413
Fat yield (%)	2.0	2.8	2.7	0.43	0.432	0.305	0.441
Liver weight (g)	50.8	51.6	48.5	4.81	0.899	0.753	0.754
Heart weight (g)	9.7	11.4	11.0	1.34	0.659	0.514	0.543
Gizzard weight without cuticle (g)	24.4	18.6	17.7	2.29	0.165	0.086	0.425
Ratio of edible parts to inedible parts	1.9	2.1	2.0	0.18	0.594	0.529	0.437

Data represent the LS means and pooled standard error of the mean. Orthogonal polynomials were used to evaluate linear and quadratic responses to the levels of saponins. Means denoted within the same row with different superscripts are significant (*p* < 0.05).

**Table 6:** Biochemical blood parameters of broiler chickens.

Indicators	Groups			SEM	*p*-Value		
	CON	SAP	SAP-FREE		Anova	Linear	Quadratic
22 days							
Total protein (g/L)	27.1	29.6	30.0	2.04	0.572	0.343	0.698
Albumin (g/L)	13.6	14.2	14.9	0.86	0.592	0.326	0.964
Globulin (g/L)	13.4	15.3	15.1	1.18	0.505	0.358	0.490
ALT (IU/L)	6.0	6.6	4.9	0.67	0.291	0.304	0.229
AST (IU/L)	171.9	156.8	163.0	8.20	0.470	0.470	0.330
Glucose (mmol/L)	13.4	13.1	13.6	0.59	0.855	0.821	0.625
Total cholesterol (mmol/L)	3.5	4.0	4.1	0.19	0.136	0.075	0.344
Triglycerides (mmol/L)	0.3	0.4	0.4	0.07	0.881	0.808	0.675
Creatinine (μmol/L)	34.3	34.7	34.3	1.74	0.984	1.000	0.862
Uric acid (μmol/L)	201.1	280.8	175.0	91.64	0.711	0.847	0.440
TCWA (mg/L)	26.0	26.6	25.5	5.13	0.989	0.954	0.897
Ceruloplasmin (mg/L)	56.0	64.7	73.0	7.21	0.319	0.147	0.986
TBA-AP (μmol/L)	2.8	2.7	3.1	0.21	0.333	0.249	0.350
CP/TBA-AP	20.3	24.0	23.2	1.87	0.388	0.312	0.353
36 days							
Total protein (g/L)	37.2	37.9	35.4	2.65	0.796	0.648	0.640
Albumin (g/L)	13.9	15.9	15.7	1.28	0.502	0.357	0.484
Globulin (g/L)	23.3	21.9	19.7	1.88	0.442	0.224	0.857
ALT (IU/L)	5.6	5.4	7.2	0.88	0.350	0.236	0.411
AST (IU/L)	206.3 ^b^	203.0 ^b^	276.4 ^a^	15.57	0.026	0.019	0.091
Glucose (mmol/L)	7.3	11.8	13.0	1.75	0.127	0.061	0.461
Total cholesterol (mmol/L)	4.1	4.2	3.9	0.26	0.771	0.593	0.651
Triglycerides (mmol/L)	0.4	0.3	0.3	0.04	0.258	0.293	0.198
Creatinine (μmol/L)	30.0 ^b^	32.8 ^ab^	34.7 ^a^	0.84	0.022	0.008	0.716
Uric acid (μmol/L)	127.9	150.4	155.8	37.48	0.859	0.618	0.859
TCWA (mg/L)	29.3	26.3	28.2	2.06	0.606	0.718	0.368
Ceruloplasmin (mg/L)	145.7	103.0	81.0	28.05	0.323	0.154	0.774
TBA-AP (μmol/L)	2.5	2.6	2.4	0.22	0.875	0.847	0.647
CP/TBA-AP	60.7	39.1	34.6	12.01	0.329	0.176	0.583

Data represent the LS means and pooled standard error of the mean. Orthogonal polynomials were used to evaluate linear and quadratic responses to the levels of saponins. Means denoted within the same row with different superscripts are significant (*p* < 0.05). TCWA—total concentration of water-soluble antioxidants; TBA-AP—active products reacting with thiobarbituric acid; CP/TBA-AP—ratio of ceruloplasmin concentration to the content of active products reacting with thiobarbituric acid.

## Data Availability

The raw data supporting the conclusions of this article will be made available by the authors without undue reservations. The data presented in this study are available upon request from the corresponding author.
